# Dark Emotions Are Not Always Bad: The Role of Emotions and Professional Training in Predicting Patterns of Engagement and Burnout Among Preschool Teachers

**DOI:** 10.3390/jintelligence14030046

**Published:** 2026-03-11

**Authors:** Chaoyi Wang, Dong Yang, Jiangbo Hu, Zhenyu Cai

**Affiliations:** 1 College of Child Development and Education, Zhejiang Normal University, Hangzhou 311231, China; cywang@zjnu.cn; 2 College of Education for the Future, Beijing Normal University, Zhuhai 519087, China; 3 Macquarie School of Education, Faculty of Arts, Macquarie University, Sydney, NSW 2109, Australia; 4 Computer-Human Interaction in Learning and Instruction (CHILI) Lab, École Polytechnique Fédérale de Lausanne, 1015 Lausanne, Switzerland

**Keywords:** preschool teachers, work engagement and burnout profiles, teachers’ emotional states, pre-service and in-service ECE training, person-centered approach

## Abstract

The engagement and burnout profiles of preschool teachers are closely linked to young children’s developmental outcomes. This study investigated engagement and burnout profiles among 529 Chinese preschool teachers in relation to their emotional states, varying experiences, and professional backgrounds. The sample predominantly consisted of early-career educators, with 47.8% aged between 21 and 30 years and 33.1% having 0–5 years of work experience. Using a quantitative cross-sectional design and latent profile analysis (LPA), this study identified four distinct profiles: slightly exhausted (48.58%), moderately burned out (18.53%), engaged (25.90%), and highly burned out (6.99%). Positive emotional states, such as enjoyment, were associated with higher work engagement, while anxiety was associated with a higher probability of belonging to burnout profiles. In contrast, perceived career success and negative emotions like anger did not significantly predict work engagement and burnout profiles. Teachers with extensive teaching experience and pre-service early childhood education (ECE) training were more likely to maintain high work engagement. This study highlights the critical role of emotional states and professional ECE training in promoting preschool teachers’ work engagement and sustainable practice, particularly among early-career teachers.

## 1. Introduction

Teaching is a profession that places significant demands on both the emotions and the intellect (Nilson, 2016). Teachers’ work engagement, characterized by vigor, dedication, and absorption in daily educational activities (Schaufeli & Bakker, 2004), is closely linked to a variety of positive cognitive, social, behavioral, and emotional outcomes for students, as well as beneficial professional outcomes for teachers (Day, 2004; D. Yang et al., 2023). Engaged teachers are typically more productive, build trusting relationships with students, employ flexible teaching strategies, and create supportive classroom environments (Cao et al., 2022; Schaufeli et al., 2002). Their capacity to engage children in meaningful learning experiences is vital for nurturing children’s cognitive, social, and emotional development (Mansfield et al., 2012; Palmer, 2017; Richardson et al., 2014). However, not all teachers consistently strive to maximize their own motivation and maintain high levels of work engagement (Ronfeldt et al., 2013). Some teachers may experience work burnout, exhibiting symptoms such as emotional exhaustion, depersonalization, and a diminished sense of personal accomplishment. Such burnout can significantly threaten their well-being and job performance, ultimately impacting children’s learning experiences (Maslach & Leiter, 2016). The impact is particularly critical in the ECE field, where children’s limited autonomy places teachers at the center of educational programs and teacher-child interactions, demanding significant physical and emotional energy from teachers to achieve positive educational outcomes (Devjak et al., 2021).

Various personal factors, such as personal characteristics (gender, age, experience) and work-related characteristics (workload, professional ECE training), have been identified as predictors of teachers’ profiles of engagement and burnout (Bing et al., 2022; Day, 2004; Nwoko et al., 2023; Salmela-Aro & Upadyaya, 2020). In addition to these factors, teachers’ emotional states have attracted increasing research attention, given their central role in professional life (De Stasio et al., 2019; Purper et al., 2023). While positive emotions (e.g., joy, love) are consistently found to enhance work engagement and motivation (Burić et al., 2020), the impact of negative emotions, often called “dark emotions”, such as anxiety and frustration, remains complex and less understood (Gkonou & Miller, 2021; L. Yang et al., 2022b; Zembylas, 2002). In this study, “dark emotions” refer to the unpleasant feelings teachers experience during work-related activities and interactions with young children. Relevant research suggests that negative emotions increase stress and exacerbate burnout, leading to disengagement (Gloria & Steinhardt, 2017). However, other studies indicate that when managed effectively, these emotions can foster reflection, build resilience, and contribute to professional growth (Hershfield et al., 2013; Tugade & Fredrickson, 2004). This contradictory perspective highlights the need to explore the mechanism by which negative emotions either hinder or enhance engagement (Gross, 2015; Fredrickson, 2001). It is essential to consider both positive and negative emotions when developing strategies to support teachers’ well-being.

Despite the widespread recognition that emotions shape teacher engagement and burnout, research in the context of Chinese preschool education remains limited. Unlike many Western countries, where the teaching workforce is more diverse in age and experience, most preschool teachers in China enter the profession directly after obtaining their undergraduate degrees (4-year university education) or associate degrees (3-year vocational college education). The associate degrees typically focus on practical vocational training in early childhood education, equipping graduates with basic teaching skills for preschool settings. Consequently, the demographic profile is notably characterized by youth and a concentration in the early-career stage, typically between 21 and 30 years old. In recent years, the curriculum for Chinese preschool education has shifted from teacher-directed activities to a child-directed and play-based approach (X. Lin et al., 2021), requiring preschool teachers to adapt their teaching practices accordingly. Many teachers struggle to fully embrace play-based learning due to limited training, making the transition particularly challenging (Bautista et al., 2023). The increased pressure on teachers due to curriculum reform, along with other job demands such as managing heavy workloads, meeting parental and administrative expectations, and balancing professional and personal life, has been shown to contribute to negative emotions, decreased job satisfaction, and higher turnover rates in high-pressure environments (H. Cheng et al., 2023; Maslach & Leiter, 2008). Despite significant development in the field of ECE, many Chinese preschool teachers choose to remain in their roles and adapt to these changes, rather than intend to leave (Liu et al., 2023). While this decision may be influenced by sociocultural factors, such as the job security offered by government-owned institutions, further investigation is needed into the intrinsic factors, particularly the emotional states of these teachers, and how these factors relate to their work engagement and burnout.

Employing a quantitative cross-sectional design, this study investigated the emotional states (enjoyment, anxiety, anger, perceived career satisfaction, and perceived career success) and their relationship to work engagement and burnout among 529 Chinese preschool teachers. A person-centered latent profile analysis (LPA) was employed to explore the relationships between these variables (Holmström et al., 2023). The study aims to identify distinct profiles of work engagement and burnout, assess the predictive role of emotional states, and examine how personal characteristics (work experience, pre- and in-service ECE professional training) are associated with teachers’ engagement and burnout profiles. Based on prior research, we hypothesize that positive emotions (e.g., enjoyment, career satisfaction) will be positively associated with higher levels of work engagement. In contrast, depending on context, negative emotions (e.g., anger, anxiety) may exert detrimental and beneficial effects. Such a study increases our cross-cultural understanding of teachers’ emotional reactions to their work under pressure. The methodology employed in this study differs from previous research that primarily relies on qualitative methods and variable-centered analyses of psychological scales (D. Yang et al., 2023) and can inspire future studies that aim to capture the characteristics of teachers’ diverse profiles from self-reported data.

## 2. Literature Review

### 2.1. Teachers’ Work Engagement and Burnout and Their Profiles

Work engagement among teachers can be understood as a meaningful and affirmative bond with their work, constituting an essential aspect of occupational well-being (Bakker et al., 2003; Schaufeli et al., 2002). Numerous studies across diverse educational settings (e.g., primary, secondary, and high school) suggest that teachers’ engagement is a critical predictor of work effectiveness and professional achievement (Abós et al., 2018; Burić & Macuka, 2018; Dhiman & Arora, 2018; Lu et al., 2018; Pepe et al., 2019). In general, work engagement refers to a positive, fulfilling, and work-related state of mind characterized by three key dimensions: “vigor, dedication, and absorption” (Schaufeli et al., 2002, p. 74). Within the framework of work engagement, vigor denotes high energy and psychological resilience at work; dedication reflects strong commitment, accompanied by feelings of significance, interest, and challenge; and absorption signifies a state of deep engrossment and concentrated focus in one’s professional activities (Bakker & Demerouti, 2007). Recent studies indicate that the absorption dimension is less central, resulting in a core conceptualization of work engagement centered primarily on vigor and dedication (Drouin-Rousseau et al., 2024).

However, teaching is widely regarded as one of the most demanding professions, and many educators experience substantial work-related burnout (W. Zhao et al., 2022). The rising prevalence of teacher burnout has raised global concerns, highlighting the need to examine both the benefits of engagement and the risks of burnout (Maslach & Leiter, 2016). According to Maslach and Leiter (2016), burnout is a chronic psychological state resulting from prolonged teaching stress (Salmela-Aro et al., 2009; Teuber et al., 2021). It is defined by three core components: exhaustion, depersonalization, and a diminished sense of personal accomplishment. Teacher burnout involves emotional fatigue and feelings of being overwhelmed by teaching demands. It often leads to a cynical attitude toward their profession, resulting in detachment from both their students and their work. Educators experiencing burnout may also feel ineffective in their roles, leading them to doubt their impact on student learning. A key symptom of burnout is emotional exhaustion, which can be triggered by factors such as high perceived workloads, intense occupational stress, negative attitudes toward teaching responsibilities, and feelings of professional inadequacy (Dhiman & Arora, 2018). As these symptoms accumulate, they can severely impact teachers’ overall well-being and teaching effectiveness.

The multidimensional nature of work burnout and engagement adds complexity to their dynamic relationships (Drouin-Rousseau et al., 2024). Recent person-oriented research indicates that teacher engagement and burnout are not merely opposing constructs but can coexist in various contexts, especially in high-demand work environments (Holmström et al., 2023; Klusmann et al., 2008). Previous studies have consistently identified at least two major profiles among teachers: the “highly engaged” profile, characterized by high levels of work engagement with minimal burnout symptoms, and the “engaged-burnout” profile, where teachers show high engagement but also experience significant burnout, especially in high-demand, low-support environments (Klusmann et al., 2008; Salmela-Aro et al., 2019. Additional profiles have been identified in certain contexts, such as the “burnout” profile, characterized by high burnout and low engagement, and the “burnout risk” profile, in which teachers experience moderate levels of both burnout and engagement (Collie et al., 2015; Salmela-Aro et al., 2009). Most teachers fall into profiles indicating some level of burnout, suggesting the prevalence of burnout symptoms even among those engaged (Salmela-Aro et al., 2019).

However, most research on teachers’ engagement and burnout has been conducted in Western cultural contexts, including Finland (e.g., Eteläpelto et al., 2015; Pyhältö et al., 2015), the Netherlands (e.g., Van der Heijden et al., 2015), North America (e.g., Fu & Clarke, 2017), and the UK (e.g., Edwards, 2005), leaving a significant gap in understanding these dynamics in Eastern educational settings (Toom & Husu, 2024). This gap is particularly notable in China, where high levels of teacher burnout and emotional exhaustion have been documented. For example, Chinese teachers’ emotional exhaustion scores are the second-highest globally (mean = 54.75, range: 15–75), significantly above the global average of 38.29 (García-Arroyo et al., 2019). Furthermore, Zheng and Guo (2017) reported that the negative association between emotional intelligence and burnout was particularly pronounced among Chinese teachers compared with international peers. Their findings suggest that stressors unique to the Chinese educational context may contribute to these higher levels of burnout.

Although burnout is highly prevalent in China, most existing studies focus on general burnout and rely heavily on traditional methods (e.g., qualitative approaches), which often overlook distinct engagement-burnout profiles, especially among Chinese preschool teachers (Li et al., 2024). For example, while Sun et al. (2022) examined the relationship between professional identity and burnout among Chinese female teachers, including 16.9% from preschools, their study did not specifically investigate unique engagement-burnout profiles within this group. As one of the countries with the largest teaching workforce and home to the world’s second-largest child population, China urgently needs more research to identify and understand engagement and burnout profiles among preschool teachers. Such research could provide valuable insights for culturally tailored interventions and support strategies, ultimately improving teacher well-being and educational quality in China.

### 2.2. The Role of Emotions in Predicting Preschool Teachers’ Engagement and Burnout Profiles

Teaching is widely acknowledged as a profession that demands both emotional depth and cognitive rigor, requiring teachers to apply intellectual skills and engage in intensive emotional management to create supportive learning environments (Hargreaves, 2000; Zhang et al., 2020). Teachers’ emotions refer to complex, multicomponent, and dynamic affective experiences that educators encounter in professional, social, and cultural contexts. Considered the “heart” of teaching, these emotions are not merely internal feelings but are actively constructed through interactions with students, colleagues, parents, and administrative policies (S. Yang et al., 2022c). These emotional demands are especially intense for preschool teachers, who often serve as both educators and caregivers (Zinsser et al., 2016). Their emotional expressions and interactions are essential for establishing a safe, nurturing environment that promotes early development and fosters positive relationships with young children (Jennings & Greenberg, 2009). The developmental needs of preschool-aged children impose an extra layer of emotional responsibility, requiring teachers to provide sustained patience, empathy, and adaptability. As a result, early childhood education (ECE) teachers frequently experience considerable emotional strain due to the high levels of emotional investment required to engage effectively with young children (C. Cheng, 2024). This underscores the critical role of emotional well-being in sustaining preschool teachers’ work engagement.

Research emphasizes that teacher emotions are not isolated psychological responses; rather, they are shaped by instructional practices and interactions with children (Frenzel et al., 2009; Hagenauer & Volet, 2014; Sutton & Wheatley, 2003; Weiner, 2007; Wentzel, 2009). Studies indicate that different teacher emotions are associated with distinct levels of work engagement. Positive emotions, such as enjoyment, satisfaction, and pride, can enhance motivation and engagement, fostering a more dynamic and supportive learning environment (Chen, 2016). In contrast, negative emotions, such as anger, frustration, and anxiety, are often linked to disengagement and burnout, potentially reducing teaching effectiveness and adversely affecting the classroom climate (Becker et al., 2011; Taxer & Frenzel, 2015).

Studies indicate that teachers frequently manage three core emotions: enjoyment, anxiety, and anger (Bach & Hagenauer, 2022; Huang et al., 2020). Enjoyment is one of the most prominent positive emotions experienced by teachers. It manifests in two primary forms: anticipatory joy, arising from looking forward to a desirable event, and activity-related joy, stemming from direct involvement in inherently satisfying tasks (Keller et al., 2014; Sutton & Wheatley, 2003). Anxiety reflects anticipatory worry about potential threats. It refers to the anticipation of future danger, and includes not only cognitive components (concerns, worries, or handling tough situations) but also physiological components (sweating, insomnia, problems with decision-making) (American Psychiatric Association, 2013). Anger is a negative emotion triggered when undesirable events are attributed to a specific responsible party (Kuppens et al., 2003). As one of the most salient negative emotions, anger is associated with undesirable teaching strategies (Frenzel et al., 2016) and negatively associated with teachers’ well-being (Taxer & Frenzel, 2015).

Enjoyment arises from engaging in fulfilling activities, while anxiety and anger tend to emerge in response to anticipated threats or unmet expectations. These two negative emotions can exert substantial psychological and physiological impacts on teachers, affecting their emotional well-being and, consequently, their professional performance (Kuppens et al., 2003). These acute emotional states differ from burnout and engagement, which involve malleable positive and negative affective responses. By contrast, burnout constitutes a chronic psychological state stemming from long-term cumulative stress.

Beyond these core emotions, recent studies have highlighted the role of broader emotional factors, such as perceived career satisfaction and perceived career success, in shaping teachers’ emotional experiences (Atmaca et al., 2020; Judge & Bono, 2001; King & Chen, 2019; Nalipay et al., 2019; Parveen & Bano, 2019). Perceived career satisfaction reflects a sustained evaluation of one’s work experiences (Greenhaus et al., 1990), while perceived career success refers to subjective appraisals of professional achievements (Eby et al., 2003). While core emotions such as enjoyment, anxiety, and anger directly influence teachers’ well-being and their classroom practices, career satisfaction and career success are increasingly recognized as integral components of the broader emotional landscape in teaching and are closely intertwined with emotional well-being. Thus, they are treated as key emotional correlations of engagement and burnout.

Research has shown that teachers who perceive themselves as successful and capable of managing classroom challenges tend to experience more positive emotions, which enhance their engagement and resilience (Atmaca et al., 2020; Nalipay et al., 2019). Conversely, teachers with a lower sense of career success are more likely to encounter negative emotions, often stemming from perceived inadequacies in their coping skills, which may lead to burnout and emotional exhaustion (Parveen & Bano, 2019). Furthermore, some research considers job satisfaction as a subcategory of emotional experience (Atmaca et al., 2020; Nalipay et al., 2019; Rodrigo-Ruiz, 2016; Schutz & Lee, 2014). For instance, King and Chen (2019) found that teachers with high job satisfaction are more likely to report positive emotions, which buffer them against the adverse effects of occupational stress. Similarly, research by Atmaca et al. (2020) and Nalipay et al. (2019) found that job satisfaction not only promotes positive emotions but also mitigates the risk of emotional exhaustion and disengagement among teachers.

Despite these insights, there remains a gap in understanding how these broader emotional factors, such as perceived career satisfaction and career success, interact with core emotions to influence teachers’ engagement and burnout, especially within the emotionally demanding context of ECE. Further research is needed to examine how these broader emotional dimensions shape teachers’ engagement and burnout profiles, providing evidence to support teachers in emotionally intensive roles.

### 2.3. The Association Between Characteristics and Engagement (Burnout) Profiles

Personal characteristics, including gender, age, teaching experience, and educational qualifications, significantly influence levels of engagement and burnout among preschool teachers (Salmela-Aro & Upadyaya, 2014). Over the past decade, the teaching workforce has increasingly been composed of bachelor’s degree graduates, who often experience “reality shock” when entering the profession. This shock arises from the gap between theoretical knowledge and practical classroom demands, as well as from ambiguous professional identity. This maladjustment period represents a critical precursor to early-career burnout and turnover intentions (Liu et al., 2023).

The level of educational qualifications and specialized pre- and in-service training emerges as a critical factor. While higher formal qualifications are often associated with greater professional competence, targeted in-service training can directly enhance teachers’ self-efficacy and resilience, thereby serving as a buffer against stress and promoting engagement (Muenchhausen et al., 2021). Evidence indicates that targeted, needs-based professional development (referred to as “menu-style”) training programs can significantly enhance teachers’ self-efficacy, professional belonging, and engagement by providing practical resources and emotional support (Zhang et al., 2020). Furthermore, in high-pressure environments, lower qualification levels have been identified as a key risk factor for turnover intention among early childhood educators (Heilala et al., 2024). By identifying how these personal and work-related characteristics intersect with engagement and burnout patterns, schools and policymakers can better tailor professional development programs and support systems to meet educators’ diverse needs.

### 2.4. The Present Study

Existing research confirms strong connections between teachers’ engagement and burnout profiles and the quality of education (Salmela-Aro et al., 2019). Teachers’ personal factors, particularly teaching experience and pre- and in-service ECE training, may significantly influence their engagement and burnout. Both positive and negative emotions are associated with their distinct positions on the spectrum of engagement and burnout. Grounded in these notions, this study aims to explore the relationships between teachers’ profiles and their emotional states, and to investigate the profiles of engagement and burnout among Chinese preschool teachers with varied work experience and educational backgrounds.

This study differs from prior research in three main aspects. First, it focuses on Chinese preschool teachers, addressing a study gap that primarily targets Western primary and high school teachers. Second, it employs person-centered LPA to identify distinct subgroups within the population, offering a nuanced understanding of engagement and burnout. Third, it explores both positive and negative emotions, including enjoyment, anxiety, anger, perceived career satisfaction, and perceived career success. These emotions were chosen because they represent an integral component of the broader emotional landscape in teaching, which was under-researched. Understanding these relationships will provide comprehensive insights into how emotional experiences shape teachers’ professional profiles in a specific cultural context.

The study addresses the following research questions:

1. What are the profiles of engagement and burnout among the preschool teachers?
2. How do different types of preschool teachers’ emotional states (enjoyment, anxiety, anger, perceived career satisfaction, and perceived career success) predict profile membership of engagement and burnout?
3. How do preschool teachers’ personal characteristics (teaching experience and pre- and in-service ECE training) predict profile membership of engagement and burnout?

## 3. Methods

### 3.1. Sample and Procedure

The sample for this study comprised 529 participants (96.2% female), all recruited from 20 public preschools in Zhejiang, a southeastern province of China. Participants were selected using purposive sampling with two key inclusion criteria: (1) all preschools were rated as first-class preschools, which is the highest level in China’s preschool evaluation system, indicating qualified teacher-student ratios and high-quality educational resources; (2) the 20 preschools were geographically distributed across five cities in Zhejiang province, including the provincial capital and four other municipal cities, to enhance sample representativeness. Within this framework, we further ensured diversity in teachers’ work experiences and pre- and in-service ECE training. All preschools were comparable in quality rating, staff-to-child ratios, and all followed standardized educational practices, with differences only in teachers’ years of service and career trajectories.

Regarding age distribution, 47.8% (*n* = 253) of the participants were between 21 and 30 years old, 44.4% (*n* = 235) were between 31 and 40 years old, and 7.8% (*n* = 41) were 41 years or older. Regarding educational qualifications, 83.9% (*n* = 444) held a bachelor’s degree, 6.8% (*n* = 36) possessed a master’s degree, and the remaining 9.3% (*n* = 49) held an associate degree from vocational colleges. Of all participants, 84.1% (*n* = 448) had graduated from ECE programs and completed pre-service training in ECE, while 15.9% (*n* = 81) majored in related fields such as psychology or the arts. For teaching experience, 33.1% (*n* = 175) had 0–5 years of experience, 30.6% (*n* = 162) had 6–10 years, 18.7% (*n* = 99) had 10–15 years, and 17.6% (*n* = 93) had more than 15 years. The median experience fell within the 6–10-year range, reflecting a diverse yet typical staffing arrangement in Chinese public preschools.

The survey consisted of demographic data from preschool teachers, covering age (e.g., 21–30 years; 31–40 years), gender (female, male), ECE training backgrounds, and teaching experiences (e.g., 0–3 years; 3–5 years; 6–10 years), as well as self-reported work engagement, burnout, career satisfaction and success, and perceived emotions. Participants were given an informed consent form before taking part in the survey. Before agreeing to proceed, they were fully informed about the study’s purpose and data management plan. On average, participants completed within 15 min during their lunch break.

This study was conducted in accordance with the Declaration of Helsinki and its later amendments. Ethical approval was obtained from the Ethics Committee of Zhejiang Normal University (Approval No. ZSRT2024130). All participants provided written informed consent before data collection.

### 3.2. Measures

To assess the constructs of interest, we adopted validated scales from the existing literature. The instruments were translated into Chinese and have been widely used in numerous empirical investigations conducted in Chinese contexts. In the present research, all scales exhibited satisfactory reliability among the Chinese sample, with Cronbach’s α coefficients for each dimension exceeded 0.75, further supporting their strong internal consistency. Furthermore, cross-sample reliability analysis revealed that the scale’s reliability remained consistent across diverse subgroups, highlighting their robustness and applicability in the Chinese cultural and educational context.

Teachers’ work engagement. Work engagement was assessed using the short form of the Utrecht Work Engagement Scale (UWES-S) (Schaufeli et al., 2006), chosen for its high validity and wide applicability in educational research. This scale consists of nine items measuring work-related vigor (three items, e.g., “*When I work*, *I feel that I am bursting with energy*”), dedication (three items, e.g., “*I am enthusiastic about my work*”), and absorption (three items, e.g., “*Time flies when I am working*”) to be rated on a 7-point scale (from 0 = “not at all” to 6 = “daily”). The items capture the emotional, cognitive, and behavioral dimensions of work engagement. We employed the overall measure developed by Schaufeli et al. (2006), computing a sum score to reflect teachers’ global work engagement. The scale demonstrated excellent internal consistency, with a Cronbach’s α of 0.95 in the present study. Its validity and reliability have been well established in Chinese samples through extensive prior use (Hu & Schaufeli, 2009).

Teachers’ work burnout. In the study, work burnout was measured with the Bergen Burnout Inventory (Salmela-Aro et al., 2009, 2011). In the original study, the inventory comprises 9 items measuring three factors of work burnout: exhaustion (“*I feel overwhelmed by my work*,” α = 0.73); cynicism about the meaning of work (“*I feel lack of motivation in my work and often think of giving up*,” α = 0.79), and inadequacy (“*I often have feelings of inadequacy in my work*,” α = 0.80). These dimensions capture the emotional, cognitive, and behavioral facets of burnout. All items were rated on a 6-point scale (1 = strongly disagree to 6 = strongly agree). Sum scores were formed to represent the different components of burnout. This inventory has been adapted for use in China and validated across various groups (Teuber et al., 2021). For the present sample, Cronbach’s α was computed separately for each dimension, revealing acceptable reliability for exhaustion (0.86), cynicism (0.88), and inadequacy (0.89). 

Teachers’ career satisfaction. Preschool teachers’ perceived career satisfaction was assessed using a five-item scale by Greenhaus et al. (1990), which captured progress toward career goals, satisfaction with job accomplishments, and alignment with personal values. Participants expressed their level of agreement with each statement (e.g., “*I am satisfied with the progress I have made toward meeting my overall career goals*”) using a 5-point Likert scale (1 = strongly disagree to 5 = strongly agree). The mean score served as an indicator of career satisfaction, with higher scores representing greater satisfaction. The scale has demonstrated good internal consistency (Cronbach’s α = 0.89) and has been validated in Chinese samples (Li et al., 2025).

Teachers’ career success. Preschool teachers’ perceived career success was assessed using the Career Success Scale (CSS; Eby et al., 2003). This scale comprises two dimensions: intra-organizational competitiveness (e.g., perceived opportunities for advancement or promotion within the current organization) and extra-organizational competitiveness (e.g., perceived ease of finding an equivalent or better job with a different employer), each with three items, for a total of six items. The 5-point Likert scoring method was used for this assessment, ranging from 1 (strongly disagree) to 5 (strongly agree). In this study, the scale achieved excellent internal consistency, with a Cronbach’s α of 0.93.

Teachers’ emotions. Teachers’ emotions were measured by the Teacher Emotion Scale. The TES includes 12 items assessing three discrete emotions: enjoyment, anxiety, and anger, experienced during teaching and interacting with children. Following a rigorous back-translation procedure, the Chinese version was validated by Huang et al. (2020). The three subscales are as follows: enjoyment (4 items; e.g., “*I generally enjoy teaching*”), anxiety (4 items; e.g., “*Preparing to teach often causes me to worry*”*)*, and anger (4 items; e.g., “*I often feel annoyed while teaching*”*).* All items were rated on a 4-point scale from 1 = strongly disagree to 4 = strongly agree. In this study, Cronbach’s α values were 0.94 for enjoyment, 0.96 for anger, and 0.94 for anxiety.

### 3.3. Analysis Plan

All analyses were conducted using Mplus (Version 8.0). Since the variables were measured using different Likert-point scales, all indicators were standardized prior to analysis. Given that participants were nested within 20 preschools, we accounted for clustering at the preschool level using the “TYPE = COMPLEX” function in Mplus. This function accounts for non-independence of observations within the same preschool by estimating robust standard errors (SE) and adjusting chi-square statistics, ensuring valid statistical inferences for both LPA and subsequent logistic regression analyses.

To identify homogeneous latent subgroups of teachers based on their levels of exhaustion, cynicism, feelings of inadequacy, and engagement, we performed LPA using the three-step approach (Asparouhov & Muthén, 2014). This approach allows examination of associations between profile membership and teacher characteristics (work experience, pre- and in-service ECE training) while controlling for measurement error in classifying individuals into latent profiles. The clustering adjustment was incorporated into the LPA model by specifying preschool as the cluster variable, ensuring that the latent class enumeration and classification were not biased by intra-preschool similarities.

Data quality was examined prior to analysis. Of the initial 533 participants, four were excluded due to incomplete responses on the core variables (i.e., the engagement and burnout scales), resulting in an effective analysis sample of *n* = 529 for all subsequent analyses, including the LPA and logistic regressions.

For RQ 1, LPA was performed on standardized indicators of work burnout (exhaustion, cynicism, and feelings of inadequacy) and work engagement, conducted in two stages. In the first stage, models specifying two to five latent profiles were estimated. Model comparison relied on several fit statistics: the Bayesian Information Criterion (BIC), the Vuong–Lo–Mendell–Rubin Likelihood Ratio Test (VLMR-LRT), the Bootstrapped Likelihood Ratio Test (BLRT), and entropy (Nylund et al., 2007). Estimation proceeded iteratively from a one-class baseline through successive k-class solutions. The optimal solution was determined by balancing empirical fit indices with theoretical interpretability.

To address RQ 2 and RQ 3, covariates, including teachers’ emotions (e.g., enjoyment, anxiety) and personal attributes (e.g., teaching experience, pre- and in-service ECE training) were introduced into the final LPA model to examine their associations with profile membership. For logistic regression analyses predicting profile membership from covariates, the “TYPE = COMPLEX” function was retained to adjust for preschool clustering, ensuring robust standard errors for odds ratios (OR). This design allowed us to examine how these variables predict or associate with latent profile membership. Covariates were added to the model in separate analyses to examine their unique associations with profile membership. Correlations, means, and variances for all the examined variables are presented in Table 1.

To further ensure the rigor of the analyses, strategies were employed to address missing data, and the choice of method was contingent on the specific analysis. The proportion of missing data was minimal, with no variable exceeding 2.5%. Given the low proportion of missing data and the absence of any discernible pattern in the observed variables, the following strategies were employed. For the LPA, we applied the Full Information Maximum Likelihood (FIML) method in Mplus, which allows the inclusion of all available data without imputing missing values, thereby providing unbiased parameter estimates. For logistic regression analyses, listwise deletion was employed, resulting in a final analytical sample of *n* = 529. This represents 99.2% of the original full sample (*n* = 533). Given that fewer than 1% of cases were excluded due to missingness, the potential bias introduced by listwise deletion is considered minimal, and the analytical samples from both procedures are comparable. Sensitivity analyses compared the descriptive statistics (means, standard deviations) of core variables (e.g., engagement, burnout, emotional variables) between the excluded cases and the retained sample. Results showed no meaningful differences between the two groups (all Cohen’s d < 0.10, indicating trivial effect sizes), further verifying that listwise deletion did not introduce substantive bias (Graham et al., 2007).

## 4. Results

### 4.1. Latent Profiles of Preschool Teachers Based on Work Engagement and Burnout

As shown in Table 2, models with one to six latent profiles were evaluated to identify the optimal solution. The fit indices suggested that the six-profile approach may better match the data, as Entropy was near 1 in this model, and the AIC, BIC, and adjusted-BIC were lower than in earlier models. The likelihood of correctly identifying teachers in each profile ranged from 0.81 to 0.88.

However, the four-profile solution was selected based on the adjusted VLMR test results, which indicated that the four-profile solution was the most appropriate model for our data, as it provided the best balance between interpretability and fit compared to the three- and five-profile solutions. While the six-profile model exhibited better fit and the highest entropy, it included a smaller profile with only 5 cases (0.94% of the total sample). The four-profile solution with a minimum profile proportion of 6.94% (37 cases) should be retained to enhance power and precision relative to the larger-profile solution. Accordingly, the four-profile solution was selected as the most parsimonious and interpretable model (Olivier et al., 2018).

**Figure 1:**
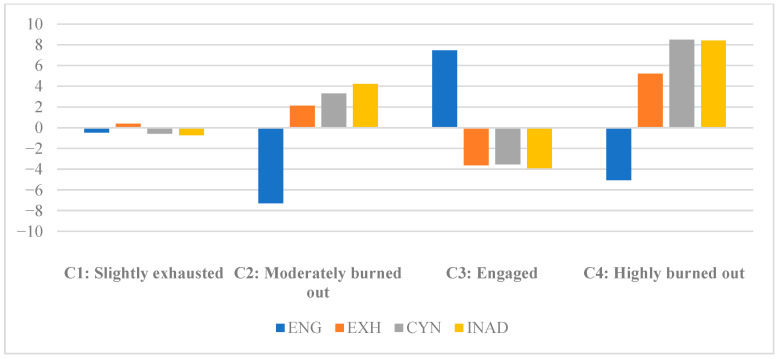
Standardized means on the clustering variables across four profiles (*n* = 529).

As illustrated in Figure 1, the four-profile solution was generated from the LPA. The first profile represented the largest subgroup comprising nearly half (48.58%; *n* = 257) of the sample and was labeled *slightly exhausted (C1)*. This profile yielded slightly below-average levels of engagement, cynicism, and inadequacy, but a slightly higher level of exhaustion relative to the sample mean. The second profile (18.53%, *n* = 98) was labeled *moderately burned out (C2)* because it exhibited a below-average level of teacher engagement (the lowest degree) and moderately exceeded the sample mean on all burnout indicators. In addition, a subgroup of nearly one-quarter (25.90%; *n* = 137) of the study participants was labeled *engaged (C3)*. This profile stood out for its relatively high level of engagement (~0.77 SD above the sample mean) and notably low levels of exhaustion, cynicism, and inadequacy (~0.94–1.00 SD below the sample mean). The fourth and smallest subgroup, containing only 6.99% (*n* = 37) of the sum, was named *highly burned out (C4)* since it manifested markedly elevated levels of exhaustion (~1.36 SD above the sample mean), cynicism (~2.39 SD above the sample mean), and inadequacy (~2.12 SD above the sample mean), while engagement was below the sample mean (~0.53 SD below the sample mean). Descriptive statistics for the unstandardized final profile solution are presented in Table 3.

### 4.2. The Role of Emotional States in Predicting Engagement Profiles Among Preschool Teachers

A follow-up logistic regression analysis was conducted to examine how teachers’ emotional states predicted membership across the four engagement–burnout profiles. In the regression model, five variables related to teaching emotions (enjoyment, anxiety, anger, career satisfaction, and career success) were included as covariates. Adding these covariates to the model resulted in a similar profile membership to that in the final LPA model, with respect to the size and interpretation of the latent groups.

In general, the results with the covariates (Table 4) indicated that preschool teachers who reported higher levels of positive emotions, particularly career satisfaction and enjoyment, were more likely to belong to the “*engaged*” profile (C3) rather than the burned-out-related profiles. Specifically, teachers reporting higher levels of satisfaction and enjoyment were more likely to be in the “*slightly exhausted*” profile (C1) compared to the “*highly burned out*” profile (C4), and more likely to be in the “*engaged*” profile (C3) than the “*highly burned out*” profile (C4).Conversely, teachers with higher levels of anxiety were more likely to belong to the *burned-out profiles* (C2 or C4) than to the “*engaged*” profile, indicating that anxiety is a strong predictor of burnout.

Interestingly, neither positive emotions, such as perceived career success, nor negative emotions, such as anger, emerged as significant predictors of profile membership. This pattern is somewhat surprising, as one might expect a strong sense of accomplishment to enhance teachers’ positive work states. The present findings suggest that a sense of accomplishment alone may not be sufficient to directly drive engagement; its effect may be overshadowed by other stronger emotions, such as enjoyment. Similarly, anger, which is typically associated with negative work outcomes, did not significantly predict teachers’ engagement or burnout profiles. This implies that, although teachers may experience anger in their roles, it may not have as direct or disruptive an effect on their professional engagement as anxiety does. In other words, anxiety appears more disruptive to work engagement, while anger does not necessarily correlate with burnout or low engagement. These findings offer a nuanced view of how different emotions uniquely influence teachers’ engagement and burnout profiles.

### 4.3. Associations Between Teachers’ Characteristics and Engagement and Burnout Profiles

As indicated in Table 5, teaching experience appears to play a role in differentiating between levels of work engagement and burnout. Specifically, more experienced teachers tend to fall into the “*engaged*” profile (C3) rather than the “*slightly exhausted*” (C1) or “*moderately burned out*” (C2) profiles. This pattern suggests that greater teaching experience may contribute to resilience and sustained engagement, helping teachers manage work-related stress more effectively and reducing the likelihood of severe burnout.

According to the odds ratios, teachers with pre-service ECE training were more likely to belong to the “*engaged*” profile (C3) than to the “*highly burned out*” (C4) or “*slightly exhausted*” (C1) profile, indicating that pre-service ECE training promotes higher engagement. Conversely, teachers without pre-service ECE training were more likely to fall into the “*moderately burned out*” profile (C2), indicating a moderate level of burnout. Interestingly, participation in in-service professional training did not significantly predict membership in any specific engagement or burnout profiles, suggesting that professional development alone may not be a strong factor in influencing engagement or reducing burnout. Taken together, these results suggest that teachers with longer teaching experience and pre-service ECE training tend to have higher engagement levels, particularly compared to those in the “*slightly exhausted*” (C1) and “*moderately burned out*” (C2) profiles. Teachers without these characteristics, particularly those without a pre-service ECE training background, may be more susceptible to moderate burnout than to high engagement. However, it is important to note that while teaching experience and pre-service ECE training are associated with engagement, they do not significantly reduce the likelihood of “*highly burned out*” (C4) in the present sample.

## 5. Discussion and Limitations

### 5.1. The Latent Profiles of Preschool Teachers’ Engagement and Burnout Profiles

Using a person-oriented approach, this study identified four distinct profiles of preschool teachers’ work engagement and burnout: slightly exhausted (48.58%), moderately burned out (18.53%), engaged (25.90%), and highly burned out (6.99%). The study further examined associations between these profiles, personal characteristics (e.g., teaching experiences, pre- and in-service ECE training) and teacher emotions. We identified four distinct profiles based on preschool teachers’ levels of work engagement and work burnout: *slightly exhausted* (C1), *moderately burned out* (C2), *engaged* (C3), and *highly burned out* (C4). The largest group, comprising 48.58% of the sample (C1), fell into the slightly exhausted profile. In total, 25.52% of teachers fell into the two burnout profiles (C2 and C4 combined), and only approximately one-quarter of teachers (25.90%) were classified as *engaged* (C3). This distribution highlights high levels of burnout among Chinese preschool teachers, consistent with prior research (Di et al., 2023; Liu et al., 2023; N. Zhao et al., 2023).

The high prevalence of burnout among Chinese preschool teachers reflects unique workload-related challenges. Unlike primary and high school teachers, who focus primarily on academic instruction, preschool teachers must balance a diverse array of responsibilities, including designing stimulating classroom environments, managing extensive paperwork, and fulfilling administrative tasks, often working an average of 9.28 h per day with 1.46 h of additional overtime (Y. Lin et al., 2017). Regional and institutional priorities further exacerbate these challenges. For example, in provinces such as Zhejiang, integrating STEM (science, technology, engineering, and mathematics) and other innovative practices into early childhood education has increased teachers’ workload, despite the intention to promote professional growth (Ye et al., 2024). These systemic demands, coupled with limited professional autonomy, create a high-pressure work environment that leads to emotional exhaustion and reduced engagement (H. Cheng et al., 2023).

Globally, teacher burnout is a widespread issue, with many countries reporting high levels of burnout among educators (Ozamiz-Etxebarria et al., 2023). However, some countries report relatively low levels of teacher burnout. For instance, Finnish preschool teachers paint a different picture, with the majority (54%) classified as engaged and only 13% experiencing burnout (Holmström et al., 2023). This stark difference may be attributed to higher levels of professional autonomy and organizational support in Finland, where teachers often have greater discretion over pedagogical practices (Schaufeli, 2018; Aho et al., 2006). Autonomy and agency are culturally embedded values that vary across contexts, shaping the concept of a “good” professional (Day et al., 2007). In Finland, self-directed decision-making and independence are central to the teaching profession, fostering an environment where engagement thrives. Research suggests that such autonomy allows Finnish teachers to craft contextually relevant and personally meaningful teaching strategies, enhancing their sense of professional fulfillment and reducing stress (Pietarinen et al., 2016; Pyhältö et al., 2021). Additionally, Finnish teachers benefit from collaborative work environments and a strong societal respect for the teaching profession, which collectively bolster job satisfaction and engagement while mitigating burnout risks (Hakanen et al., 2006).

Conversely, in China, systemic and organizational demands are significantly higher, and professional autonomy is often limited, contributing to heightened stress and burnout levels among teachers. Chinese preschool teachers frequently face a rigid curriculum and high administrative expectations, which constrain their ability to exercise professional discretion (Zheng & Guo, 2017). The intense workload, combined with the cultural emphasis on academic achievement and performance metrics, creates a high-pressure work environment that often exacerbates emotional exhaustion and reduces engagement (H. Cheng et al., 2023). Moreover, while Finnish teachers benefit from supportive and collaborative networks, Chinese teachers report less access to peer support and emotional resources, further increasing their risk of burnout (Y. Lin et al., 2017; N. Zhao et al., 2023). These findings underscore the critical need for tailored strategies to address the systemic and cultural challenges faced by Chinese preschool teachers and highlight the importance of fostering a supportive and collaborative work environment to mitigate burnout.

### 5.2. The Significant Role of Emotions in Teachers’ Work Engagement and Burnout Profiles

There is ample evidence that teachers’ emotions are associated with work engagement (Burić & Macuka, 2018) and that positive emotions protect against school burnout (D’Amico et al., 2020; Salmela-Aro et al., 2008). As expected, the present study found that preschool teachers with a higher level of career satisfaction and enjoyment were more likely to belong to the engaged profile. This finding is consistent with previous studies (Burić & Macuka, 2018; D’Amico et al., 2020; Greenier et al., 2021). For instance, Burić and Macuka (2018) used a longitudinal design to investigate teachers’ emotions and work engagement. Teachers who initially experienced more positive emotions (e.g., joy) tended to show greater work engagement later. This finding is also supported by studies in EFL settings, where positive emotions promote teacher engagement, whereas negative emotions such as anxiety and fatigue are associated with lower engagement (Burić & Macuka, 2018).

Additionally, we found that the participating teachers who reported higher levels of negative emotions were more likely to belong to the burnout profile than the engaged profile. This finding aligns with the research that teachers who express higher levels of anxiety are more likely to exhibit high emotional exhaustion and depersonalization, which are the core components of burnout (Kamtsios & Kakouris, 2025; Maslach & Leiter, 2016). Teachers experiencing chronic anxiety invest substantial cognitive and emotional resources devoted to threat vigilance and worry, which directly drains their vigor and dedication (Schaufeli et al., 2002). Such resource depletion impairs their capacity for effective emotional regulation, a difficulty that is itself a hallmark of anxiety disorders (Gloria & Steinhardt, 2017). As their emotional regulatory capacity diminishes, feelings of tension and inadequacy escalate, further intensifying emotional exhaustion and cynicism and reinforcing their membership in a burnout profile (Slišković et al., 2019). This pattern is particularly pronounced in high-demand contexts. Research indicates that during periods of intense stress, such as curriculum reforms or crises like the COVID-19 pandemic, teachers with higher anxiety are disproportionately likely to transition into “extreme burnout” profiles, whereas those with stronger coping strategies and self-efficacy tend to maintain engagement (S. Y. Cheng et al., 2023; Ozamiz-Etxebarria et al., 2023). Our sample, drawn from a Chinese preschool system undergoing significant pedagogical shifts and facing high workloads, likely reflects such a high-demand environment, which helps explain the strong predictive power of anxiety for burnout in our findings.

Although anger did not emerge as a significant predictor of profile membership in our sample, previous research suggests its role may be more complex and context-dependent (Burić & Frenzel, 2023; Pekrun, 2006). Anger is the most frequently encountered negative emotion among teachers, and its effects on their professional development are mixed. Slišković et al. (2019) explored how emotions mediated the relationship between principal support and work engagement among primary school teachers, finding that teachers who reported higher levels of anger were simultaneously less engaged, and vice versa. However, J. Yang et al. (2022a) found that anger is associated with a superficial engagement profile, whilst positive emotions (e.g., happiness) are a driving force behind substantial engagement. Moreover, studies have found that anger can be stimulating and is accompanied by high perceived coping potential or controllability (Pekrun, 2006). Teachers who experience anger are therefore likely to seek change and adopt an active approach to problem-solving (Burić & Frenzel, 2023). This nuanced view suggests that anger, sometimes classified as a “dark emotion”, is not inherently detrimental; rather, its impact depends on the surrounding conditions and teachers’ perceived coping potential.

Overall, our findings suggest that both positive and negative emotions play complex roles in shaping teachers’ engagement. Positive emotions (e.g., satisfaction, enjoyment) generally foster engagement and protect against burnout; the role of negative emotions (e.g., anxiety, anger) is less clear. Anxiety emerged as a risk factor for burnout, particularly in high-demand settings, whereas anger presents a more nuanced picture.

### 5.3. Associations Between Teachers’ Experience and Professional Development and Engagement and Burnout Profiles

To better understand the factors influencing teachers’ engagement and burnout, we examined how personal characteristics predicted their profile membership. Specifically, teachers with longer teaching experience were more likely to belong to the “*engaged*” profile than to the “*moderately burned out*” or “*slightly exhausted*” profiles. This pattern may reflect that greater experience often enables teachers to develop effective coping strategies, build confidence in their teaching abilities, and form a deeper connection to their work, all of which contribute to higher engagement and job satisfaction while reducing the risk of burnout (Agyapong et al., 2022). These seasoned teachers may also have cultivated a sense of accomplishment and intrinsic motivation, further supporting their resilience against burnout (Kariou et al., 2021).

Pre-service ECE training also emerged as a significant predictor. Teachers with pre-service ECE training were more likely to be in the engaged profile than those without specialized ECE training. Specialized pre-service ECE programs equip these teachers with essential skills, such as understanding young children’s developmental needs, emotional regulation techniques, and age-appropriate pedagogical strategies (Ansari et al., 2022). This specialized knowledge enhances teachers’ confidence and classroom efficacy, which are strong predictors of engagement (Xing et al., 2022). In China, pre-service ECE programs often emphasize socio-emotional learning and resilience-building, providing teachers with tools to manage the high emotional demands of their work environment. This foundation enables pre-service preschool teachers to develop a strong sense of professional identity and efficacy, which are linked to well-being and job fulfillment, and ultimately to greater effectiveness and lower risk of burnout (Lipscomb et al., 2022; Tayama et al., 2018).

Interestingly, our analysis revealed that participation in in-service training did not significantly predict teachers’ engagement or burnout profiles. This suggests that while a foundation in ECE or substantial teaching experience may enhance engagement, simply attending professional development programs may not have the same effect. One possible explanation is that many existing in-service programs may not be sufficiently tailored to address the specific emotional and pedagogical challenges faced by preschool teachers (Erol & İvrendi, 2025). Alternatively, such in-service ECE training may focus more on technical skills than on building emotional resilience and classroom management capabilities, which are critical for preventing burnout. This suggests that future in-service professional development could be designed to be more comprehensive, integrating technical skill instruction with support for emotional well-being. Therefore, understanding the complex factors influencing engagement and burnout across different teacher characteristics may support the design of more targeted, effective strategies to promote teachers’ well-being and professional effectiveness throughout their careers.

### 5.4. Limitations and Further Directions

This study has some notable limitations. First, the scope of this research was limited to exploring the association between emotional states and engagement and burnout profiles using cross-sectional data. Future research adopting other methods, such as teacher interviews or observations of teachers’ practices, could provide more detailed information to explore the type and quality of teaching experience, psychological resilience, and related factors. Specifically, longitudinal studies that document teachers’ emotional development and professional growth may provide stronger evidence. As Drouin-Rousseau et al. (2024) indicated, longitudinal studies on job engagement and burnout are essential for a more comprehensive understanding of these phenomena. Although the use of self-report instruments is appropriate for measuring perceived emotional experiences, it is important to acknowledge that engagement and burnout are complex, dynamic processes influenced by a range of social and psychological factors (Lawson & Lawson, 2013). Future research should delve deeper into these dynamics by incorporating additional factors, such as socio-emotional skills, perseverance, and academic resilience, to provide a more holistic account.

Second, while we addressed data clustering at the preschool level, the current study did not explore institutional-level factors (e.g., school culture, leadership style) and individual-level factors (e.g., teachers’ emotion regulation skills, personal characteristics) that may moderate the relationships between emotions and engagement-burnout profiles. Such factors likely interact with emotions and personal characteristics to shape teachers’ well-being. Therefore, future studies should consider these contextual elements to better understand how to create supportive environments that foster engagement and prevent burnout among preschool teachers. Multilevel research could incorporate both individual- and institutional-level variables to provide a more comprehensive understanding of contextual influences.

Third, we applied LPA in this study. However, other theoretically plausible profiles (e.g., disengaged or detached teachers characterized by low engagement and low burnout) may exist but were not captured by the variables included in this study. Future research could incorporate additional constructs, such as professional detachment, work alienation, or cynicism, to identify a broader range of teacher profiles.

Fourth, while much research has examined teachers’ anger toward students and its effects on work engagement (O’Neal et al., 2023; Rivers et al., 2007), fewer studies have examined the role of negative emotions in the bidirectional teacher-student relationship. Research in authentic classroom settings is needed to reveal the flow of emotions between teachers and students, highlighting how these emotions influence engagement and burnout across both parties and contribute to the overall educational climate.

Lastly, it is worth noting the high correlation (r = 0.824) between anxiety and anger observed in this study. This magnitude suggests substantial overlap between these two negative emotional experiences among teachers, raising questions about their empirical distinctiveness in the current context. Future research could employ confirmatory factor analysis to formally test whether teachers distinguish between anxiety and anger as separate constructs, or whether they reflect a broader dimension of negative emotionality.

## 6. Conclusions

Using a sample of Chinese preschool teachers, this study employed a person-centered LPA to investigate teachers’ work engagement and burnout. It also examined how teachers’ characteristics, such as personal traits and emotional experiences (both positive and negative, or “dark emotions”), influence their engagement and burnout patterns. Our findings further confirm that pre-service ECE training, as a critical professional background factor, significantly predicts teachers’ membership in the “engaged” profile, while in-service training alone shows limited predictive power.

Specifically, we found that teachers who experience higher levels of positive emotions, such as enjoyment and satisfaction, are more likely to belong to the engaged profile and less likely to belong to the burned-out profile. For negative emotions, anxiety is a strong predictor of burnout and is negatively correlated with engagement. Notably, anger was not a statistically significant predictor of preschool teachers’ engagement or burnout profiles in our models; its role remains unclear and may depend on moderators not measured in the present study. Taken together, these findings underscore the importance of acknowledging both the positive and negative emotional experiences teachers undergo, particularly in high-stress environments.

Importantly, this study challenges the notion that negative emotions are inherently detrimental by highlighting the nuanced roles they may play. This finding expands the current discourse by recognizing that *dark emotions are not always bad*; rather than being uniformly detrimental, their impact may be shaped by contextual or individual factors. Moreover, the findings suggest that teachers’ personal characteristics are crucial for maintaining a balanced approach to work engagement and burnout prevention. Supporting teachers in understanding and managing both positive and negative emotional experiences could play a key role in enhancing their professional resilience and reducing burnout risk.

## Figures and Tables

**Table 1:** Pearson’s correlations among all variables (*n* = 529).

Variable	1	2	3	4	5	6	7	8	9
1. ENG	—								
2. EXH	−0.295 **	—							
3. CYNI	−0.439 **	0.600 **	—						
4. INAD	−0.447 **	0.531 **	0.821 **	—					
5. SATI	0.555 **	−0.203 **	−0.261 **	−0.332 **	—				
6. SUCC	0.467 **	−0.113 **	−0.162 **	−0.259 **	0.705 **	—			
7. ENJOY	0.557 **	−0.161 **	−0.313 **	−0.302 **	0.511 **	0.415 **	—		
8. ANXI	−0.244 **	0.359 **	0.569 **	0.518 **	−0.156 **	−0.098 *	−0.191 **	—	
9. ANGR	−0.226 **	0.292 **	0.552 **	0.500 **	−0.132 **	−0.063	−0.151 **	0.824 **	—
Mean	51.960	11.050	7.210	7.780	20.040	22.650	13.290	8.170	7.480
SD	9.650	3.850	3.550	3.970	3.710	4.720	2.240	2.970	2.870
Skewness	−0.919	−0.152	1.000	0.741	−0.656	−0.109	−0.614	0.536	0.895
Kurtosis	1.041	−0.648	0.777	−0.249	0.779	0.036	1.568	0.258	1.156

Note. * *p* < .05; ** *p* < .01. ENG = Work Engagement; EXH = Exhaustion; CYNI = Cynicism; INAD = Inadequacy; SATI = Perceived Career Satisfaction; SUCC = Perceived Career Success; ENJOY = Enjoyment; ANXI = Anxiety; ANGR = Anger.

**Table 2:** Fit indices for the compared mixture models (*n* = 529).

No. of Profiles	FP	LL	AIC	BIC	SABIC	Entropy	VLMR(*p*)	BLRT(*p*)	Smallest Profile Proportion (%)
1	8	−6359.517	12,735.035	12,769.263	12,743.869	—	—	—	—
2	13	−6026.974	12,079.948	12,135.568	12,094.302	0.879	0.000	0.000	25.70%
3	18	−5903.493	11,842.986	11,920.000	11,862.862	0.811	0.253	0.000	12.57%
4	23	−5824.897	11,695.793	11,794.199	11,721.190	0.829	0.025	0.000	6.94%
5	28	−5779.704	11,615.408	11,735.207	11,646.326	0.874	0.069	0.000	2.81%
6	33	−5746.142	11,558.285	11,699.476	11,594.724	0.883	0.001	0.000	0.94%

*Note.* FP = Free Parameters, LL = Log Likelihood Value, VLMR(*p*) = *p* Value for VLMR Test, BLRT(*p*) = *p*-value for BLRT Test.

**Table 3:** Unstandardized final latent profile solution (*n* = 529).

	Engagement		Exhaustion		Cynicism		Inadequacy	
	M	SD	M	SD	M	SD	M	SD
C1: Slightly exhausted (*n* = 257)	51.49	7.79	11.42	3.06	6.62	1.52	7.05	2.02
C2: Moderately burned out (*n* = 98)	44.65	9.40	13.17	2.37	10.50	1.62	11.99	1.89
C3: Engaged (*n* = 137)	59.43	5.10	7.42	3.32	3.65	0.98	3.85	1.34
C4: Highly burned out (*n* = 37)	46.89	14.59	16.27	1.69	15.70	1.93	16.19	1.70

*Note.* M = Mean, SD = Standard Deviation.

**Table 4:** Odds ratios (OR) for emotions predicting latent profile membership (*n* = 529).

	C1 vs. C4	C2 vs. C4	C3 vs. C4	C1 vs. C3	C2 vs. C3	C1 vs. C2
	OR (95% CI)	OR (95% CI)	OR (95% CI)	OR (95% CI)	OR (95% CI)	OR (95% CI)
SATI	1.197 * (1.01, 1.41)	1.094 (0.93, 1.29)	1.455 *** (1.20, 1.76)	0.823 *** (0.74, 0.92)	0.752 *** (0.65, 0.87)	1.094 (0.99, 1.21)
SUCC	0.896 (0.77, 1.04)	0.865 (0.75, 1.00)	0.913 (0.78, 1.07)	0.981 (0.92, 1.05)	0.947 (0.86, 1.05)	1.036 (0.95, 1.13)
ENJOY	1.271 * (1.02, 1.58)	1.142 (0.91, 1.43)	1.634 *** (1.27, 2.10)	0.778 *** (0.68, 0.89)	0.699 *** (0.58, 0.84)	1.113 (0.97, 1.28)
ANXI	0.634 *** (0.49, 0.82)	0.845 (0.65, 1.09)	0.550 *** (0.41, 0.73)	1.152 (0.99, 1.34)	1.535 *** (1.26, 1.87)	0.750 *** (0.64, 0.87)
ANGR	0.852 (0.68, 1.07)	0.906 (0.72, 1.14)	0.767 (0.59, 1.00)	1.110 (0.95, 1.30)	1.181 (0.97, 1.44)	0.940 (0.81, 1.09)

*Note*. * *p* < .05; ** *p* < .01; *** *p* < .001. Groups printed in italics are the reference groups. C1 = “slightly exhausted”; C2 = “Moderately burned out”; C3 = “Engaged”; C4 = “Highly burned out”. SATI = Perceived Career Satisfaction; SUCC = Perceived Career Success; ENJOY = Enjoyment; ANXI = Anxiety; ANGR = Anger.

**Table 5:** Odds ratios (OR) for teacher characteristics predicting latent profile membership (*n* = 529).

	C1 vs. C4	C2 vs. C4	C3 vs. C4	C1 vs. C3	C2 vs. C3	C1 vs. C2
	OR (95% CI)	OR (95% CI)	OR (95% CI)	OR (95% CI)	OR (95% CI)	OR (95% CI)
Teach_exp	0.749 (0.31, 1.84)	0.563 (0.23, 1.41)	1.439 (0.52, 4.02)	0.521 * (0.29, 0.94)	0.391 * (0.18, 0.84)	1.331 (0.74, 2.38)
IST_ECE = 1	1.010 (0.41, 2.47)	0.735 (0.29, 1.84)	0.783 (0.28, 2.16)	1.290 (0.73, 2.30)	0.939 (0.44, 1.99)	1.375 (0.77, 2.44)
PST_ECE = 1	2.524 (0.76, 8.43)	1.283 (0.36, 4.54)	3.897 * (1.03, 14.73)	0.648 (0.34, 1.25)	0.329 * (0.14, 0.80)	1.968 (0.99, 3.91)

*Note.* * *p* < .05. Groups printed in italics are the reference groups. C1 “Slightly exhausted”; C2 “Moderately burned out”; C3 “Engaged”; C4 “Highly burned out”. Teach_exp = Teaching Experience (continuous variable); IST_ECE= In-service ECE training (1 = received, 0 = not received); PST_ECE = Pre-service ECE Training (1 = received, 0 = not received).

## Data Availability

The original contributions presented in this study are included in the article. Further inquiries can be directed to the corresponding author(s).
